# Impact of Desensitization on Antiviral Immunity in HLA-Sensitized Kidney Transplant Recipients

**DOI:** 10.1155/2017/5672523

**Published:** 2017-02-06

**Authors:** Mieko Toyoda, Bong-Ha Shin, Shili Ge, James Mirocha, David Thomas, Maggie Chu, Edgar Rodriguez, Christine Chao, Anna Petrosyan, Odette A. Galera, Ashley Vo, Jua Choi, Alice Peng, Joseph Kahwaji, Stanley C. Jordan

**Affiliations:** ^1^Transplant Immunology Laboratory, Cedars-Sinai Medical Center, Los Angeles, CA, USA; ^2^Biostatistics Core, Cedars-Sinai Medical Center, Los Angeles, CA, USA; ^3^Comprehensive Transplant Center, Cedars-Sinai Medical Center, Los Angeles, CA, USA

## Abstract

Viral infections represent significant morbidity and mortality factors in kidney transplant recipients, with CMV, EBV, and BKV infections being most common. Desensitization (DES) with IVIg and rituximab with/without plasma exchange followed by kidney transplantation with alemtuzumab induction increased successful transplant rates in HLA-sensitized patients but may represent an increased risk for viral infections due to severe lymphocyte depletion. Here, we report on the posttransplant viral infection status in 372 DES versus 538 non-DES patients. CMV and EBV viremia were significantly lower in DES patients, while BKV viremia was similar. This trend was observed primarily in CMV sero(−), EBV sero(+), and sero(−) patients. No patient developed PTLD. The incidence of BKAN, allograft, and patient survival was similar in both groups. These viral infections were not associated with subsequent allograft rejection which occurred within 6 months after the infection.* Conclusions.* The IVIg + rituximab desensitization combined with alemtuzumab induction with triple immunosuppression maintenance does not increase the risk for CMV, EBV, and BKV infections. Possible factors include, in addition to posttransplant antiviral prophylaxis and PCR monitoring, presence of memory T cells and antibodies specific to CMV and likely EBV, NK cell-mediated ADCC despite lymphocyte depletion, elimination of EBV and CMV reservoirs by rituximab and alemtuzumab, and use of IVIg with antiviral properties.

## 1. Introduction

Viral infections represent significant morbidity and mortality factors for immunocompromised transplant recipients [1, 2]. Cytomegalovirus (CMV) and Epstein-Barr virus (EBV) infections are common and have long been associated with significant morbidity in the renal transplant population [1–5]. Polyomavirus BK (BKV) also emerged as an important viral infection associated with risk for allograft loss. [6, 7]. The most common manifestations of CMV infection include flu-like or mononucleosis-like syndromes, leukopenia or thrombocytopenia, infection of native tissues resulting in pneumonia, gastroenteritis, retinitis, and central nerve system disease [4]. Posttransplant lymphoproliferative disorder (PTLD) is one of the most serious complications in transplant recipients and is usually associated with EBV infection [3, 8]. PTLD is a consequence of the failure of the host's immune system to contain EBV-infected B cells, resulting in uncontrolled proliferation. BKV establishes latency in the uroepithelium and persists in the renal tubules without causing disease in immunocompetent individuals [9, 10]. However, BKV reactivation occurring in renal transplant recipients may cause an acute tubulointerstitial nephritis and ureteral stenosis, leading to severe allograft dysfunction and graft loss [6, 7, 11].

We have shown that desensitization (DES) with intravenous immunoglobulin (IVIg) and rituximab with/without plasma exchange (PLEX) followed by a kidney transplantation with alemtuzumab induction increased successful transplant rates in HLA-sensitized (HS) patients [12–15]. We have also shown acceptable outcomes in patients who received ABO incompatible transplants after the modified DES protocol with IVIg, rituximab, and PLEX [12]. However, profound and prolonged B cell and T cell depletion may result in an increased risk for viral infections [16–22]. To address this, all these patients receive antiviral prophylaxis posttransplant and extensive viral-PCR monitoring to minimize viral infections and their associated complications by early detection and intervention. We have previously shown that DES patients do not exhibit a significant increased risk for viral infection compared to non-DES patients [15, 23–26], except for a significantly higher BKV infection rate in DES patients [27]. In this study, we investigated the status of CMV, EBV, and BKV viral infection and their associated complication in a much larger cohort of patients who received DES and the results were compared with those without DES (non-DES). We also investigated the impact of viral infection on allograft rejection, since an association has been suggested that viral infections may increase this risk through direct effects on allograft-directed immune responses or due to reduced immunosuppression at time of infections. [28–30]. Here, we found significantly lower CMV and EBV infection rates in DES patients and similar BKV infection rates. We then investigated patient and graft survival and immune factors possibly responsible for these findings.

## 2. Materials and Methods

This study was approved by the Institutional Review Board at Cedars-Sinai Medical Center (IRB numbers Pro00017197, 10969, and 12562). The study was conducted in accordance with the ethical guideline based on federal regulations and the common rule. CSMC also has a Federal Wide Assurance.

### 2.1. Patient Population and Healthy Volunteers

CMV, EBV, and/or BKV-PCR results in a total of 3614 and 5113 DNA samples obtained from 372 DES and 538 non-DES patients, respectively, were compared. We also examined graft and patient survival, pretransplant viral serological status, virus-associated complication, and allograft rejection. Patients examined were transplanted between January 2007 and April 2015 at Cedars-Sinai Medical Center with patient demographics shown in Table 1. Patients who were <18 years old, were monitored for viral-PCRs <2.9 months after transplant, or had <3 DNA samples obtained during the viral-PCR monitoring period (median 8.0 DNA samples per patient during median 18.7 months after transplant) were excluded.

Of 372 DES patients, 314 (84.4%) received an ABO compatible and 58 (15.6%) an ABO incompatible kidney transplant after DES. The DES protocols used for ABO compatible transplant in HS and ABO incompatible transplant in non-HS patients have been reported [12]. Briefly, a standard protocol for HLA-DES consisted of 2 doses of IVIg (2 g/kg) one month apart with one dose of rituximab (1 g) in between. The protocol for ABO incompatible transplant consisted of one dose of rituximab (1 g) two weeks prior to initiation of 5–7 sessions of PLEX followed by one dose of IVIg (2 g/kg). The combination of both protocols was used for HS patients who received an ABO incompatible transplant. If a negative or acceptable crossmatch was achieved and/or the antiblood group titer became ≤1 : 8 after DES, patients proceeded to transplantation [14, 15].

Most patients received induction therapy with lymphocyte depleting agent (alemtuzumab or anti-thymocyte globulin) or anti-IL-2 receptor antibody (anti-IL-2R, daclizumab, or basiliximab). Maintenance immunosuppression consisted of calcineurin inhibitor (tacrolimus or cyclosporine A), mycophenolate mofetil (MMF), and steroids. The target levels were dependent on the type of induction as reported elsewhere [27].

All patients received antiviral prophylaxis with ganciclovir (1.25 mg/kg daily) while inpatient and then valganciclovir or acyclovir posttransplant depending on a risk for viral infection. For transplants with CMV R−/D+, 900 mg valganciclovir was given daily for 6 months regardless of induction type. For those with lymphocyte depletion induction, 450 mg valganciclovir was given daily for 6 months for CMV R+/D+, R+/D−, or R−/D−. For those with anti-IL-2R induction, 800 mg acyclovir was given 4x a day for CMV R+/D+ or R+/D− and 800 mg daily for CMV R−/D− for 3–6 months, with dose adjustment for renal function and/or white blood cell count. CMV-, EBV-, and/or BKV-PCR monitoring was performed at 1, 2, 3, 6, 9, 12, 18, and 24 months after transplant or as needed as previously reported [23], and every two weeks in those who developed viremia. CMV and EBV infections were treated with reduction of immunosuppression in conjunction with valganciclovir (900 mg twice daily for 14 to 21 days, regardless of infection during or after antiviral prophylaxis with dose adjustments for renal function and/or white blood cell count). BKV was treated with reduction of immunosuppression, leflunomide, and/or IVIg. Antibody-mediated (ABMR) and cell-mediated (CMR) rejections were diagnosed based on the Banff 2013 [31, 32] and Banff 1997 classification [33], respectively. ABMR was treated with pulse steroids, IVIg and rituximab with or without PLEX, and CMR with pulse steroids. Refractory or Banff 2a rejection was treated with ATG.

Of 372 DES patients, 36 were monitored for lymphocyte subset analysis before and after transplant by flow cytometry, and archived sera obtained from another 38 patients were tested for total IgG and anti-EBV-IgG before and after transplant by ELISA.

Heparinized-peripheral blood samples from 20 normal adult volunteers (7 males) were tested for CMV- and EBV-specific T cell and NK cell activity.

### 2.2. Viral-PCR Assays

Viral-PCR was performed at the Transplantation and Immunology Laboratory, Cedars-Sinai Medical Center [23, 27, 34]. Briefly, for CMV- and EBV-PCR, total DNA was extracted from blood leukocytes by Qiacube (Qiagen, Valencia, CA) followed by optical density measurement, and 500 ng total DNA was submitted for the real time CMV- and EBV-PCR. CMV and EBV > 5 copies/PCR (500 ng total DNA) were considered viremia. For BKV-PCR, total DNA was extracted from 200 *μ*l of plasma and eluted in 100 *μ*l of Tris-EDTA; 10 *μ*l of the DNA solution was used for the real time BKV-PCR [27]. The result was expressed as BKV DNA copies/ml plasma and >250 copies/ml was considered viremia. Specific primers and probe used were as follows: for the CMV-PCR, specific to the CMV immediate-early antigen region (5′-CAA GCG GCC TCT GAT AAC CA-3′, 5′-ACT AGG AGA GCA GAC TCT CAG AGG AT-3′, 5′-FAM-TGC ATG AAG GTC TTT GCC CAG TAC ATT CT-BHQ-3′) [35], for the EBV-PCR, specific to the BALF5 gene encoding the viral DNA polymerase of human EBV (5′ CGG AAG CCC TCT GGA CTT C 3′, 5′ CCC TGT TTA TCC GAT GGA ATG 3′, 5′ FAM-TGT ACA CGC ACG AGA AAT GCG CC-BHQ 3′) [36], and for the BKV-PCR, specific to the large T antigen of human BKV (5′-AAA GTC TTT AGG GTC TTC TAC CTT TCT TT-3′, 5′-GAG TCC TGG TGG AGT TCC TTT AAT-3′, 5′-FAM-AAT CTG CTG TTG CTT CTT CAT CAC TGG CA-BHQ-3′) and designed by our laboratory.

### 2.3. Lymphocyte Cell Subset Analysis

The CD4+, CD8+ T cell, CD19+ B cell, and CD56+/CD16+ NK cell numbers were monitored for DES patients before and after transplant by flow cytometry using a standard 6-color direct staining method as previously described with minor modification [37, 38]. Briefly, 5 *μ*l each of the fluorochrome-conjugated antibodies to CD45 (Horizon V500, BD Biosciences, San Jose, CA), CD3 (FITC, Invitrogen), CD8 (Horizon V450, BD Biosciences), CD56 (APC, BD Biosciences), CD16 (PerCP-Cy5.5, eBioscience, San Diego, CA), and CD19 (PE-Cy7, eBioscience) was added to 100 *μ*l of heparinized blood. CD45+ cells were first separated and then plotted against forward/side scatter to separate lymphocytes. Lymphocytes were then plotted against CD3 and CD8, CD3− population was further plotted against CD19 to enumerate CD19+ B cell number, and the remaining cells plotted against CD16 and CD56. CD56+/CD16+, CD56+/CD16−, and CD56−/CD16+ were considered NK cells (CD56+/CD16+ cells). CD3+/CD8− cells were considered as CD4+ cells.

### 2.4. CMV- or EBV-Specific T Helper (CMV- or EBV-Th) and NK (CMV- or EBV-NK) Cell Analysis

CMV- or EBV-Th and NK cell levels were measured by intracellular cytokine flow cytometry (CFC) developed in our lab and described elsewhere with minor modification [39–41]. Briefly, whole blood was incubated with sucrose density purified CMV or EBV viral lysate (Advanced Biotechnologies, Eldersburg, MD) at the final concentration of 1 *μ*g/ml, together with brefeldin A and anti-CD28/CD49d overnight. After cells were stained with fluorochrome-conjugated antibodies to CD45 (V500), CD3 (FITC), CD4 (PerCP-Cy5.5), CD8 (V450), and CD56 (APC) and then with PE-anti-IFN*γ* antibody for intracellular IFN*γ* staining, followed by cell acquisition, the IFN*γ*+ cell% in CD4+ T cells and CD56+ NK cells were enumerated and defined as CMV-Th or EBV-Th and CMV-NK or EBV-NK, respectively. CMV-Th ≥ 0.20%, EBV-Th ≥ 0.10%, and CMV- and EBV-NK ≥ 0.5% were considered positive as established based on the levels detected in CMV or EBV sero(+) and sero(−) normal individuals and transplant recipients [37, 39, 40]. Phytohemagglutinin (PHA) at the final concentration of 1 *μ*g/ml was used as positive control for each sample tested. In a separate experiment where degranulation in CMV- or EBV-T and NK cells was assessed, PE-Cy7-conjugated anti-CD107a antibody was also added, and the IFN*γ*+ cell% and CD107a+ cell% in CD4+ T cells and CD56+ NK cells were enumerated. To assess the involvement of anti-CMV antibody in NK cell activation, whole blood was first incubated with IdeS (Hansa Medical, Sweden), an IgG-degrading enzyme of* S. pyogenes*, that cleaves 4 human IgG subclasses at the hinge region of IgG heavy chains, critical for ADCC [42], at the final concentration of 10 *μ*g/ml at 37°C for 1 hour, and then incubated with CMV lysate to continue the above CFC procedure.

### 2.5. Total IgG- and Anti-EBV IgG-ELISA

Total IgG (Human IgG-ELISA, Bethyl Laboratories, Inc. Montgomery, TX) and anti-EBV IgG levels (EBV-VCA IgG-ELISA, Calbiotech, El Cajon, CA) were measured by ELISA following the manufacturers' instruction. In the total IgG-ELISA, the results were expressed as mg/ml, and the levels >7, 4–7 and <4 mg/ml were considered normal, mild, and severe hypogammaglobulinemia [43], respectively. In the anti-EBV IgG-ELISA, the results were expressed as anti-EBV IgG index and the index <0.25 was considered EBV sero(−).

### 2.6. Statistical Analysis

We compared the results in the DES versus non-DES groups, viral sero(+) versus sero(−) groups, or different antiviral prophylaxis groups (Tables 1–6). Continuous variables were analyzed using Student's *t*-test and categorical variables were analyzed by Chi-square or Fisher's exact test unless otherwise stated. Total IgG and anti-EBV IgG levels before DES versus 12 months after transplant (Figure 4) and IFN*γ*+ or CD107a+ cell% in CD4+ T or CD56+ NK cells between conditions (Figure 6) were compared by paired *t*-test. The rates of CMV, EBV, or BKV viremia, allograft rejection, allograft loss, and patient death were estimated by the Kaplan-Meier method and the group differences were assessed by the log-rank test. The *p* value < 0.05 was considered statistically significant.

## 3. Results and Discussion

### 3.1. Baseline Characteristics

Baseline characteristics in DES and non-DES patients are shown in Table 1. All 372 DES patients showed PRA > 10% and 193 of those (52%) had PRA > 80% before DES. Among DES patients, 314 (84%) received DES for HLA incompatibility, while 41 (11%) and 17 (5%) received DES for ABOi or ABOi + HLA incompatibilities, respectively. There were significantly more females (61% versus 29%, *p* < 0.001), more lymphocyte depletion induction (86% versus 49%, *p* < 0.001), and maintenance with tacrolimus (99% versus 92%, *p* < 0.001) in the DES group. More female patients are HLA-sensitized due to pregnancy, and lymphocyte depletion induction and tacrolimus were used as a standard posttransplant immunosuppressive regimen for DES patients. It should be noted that 96% of DES patients induced with a lymphocyte depleting agent received alemtuzumab, while 81% of non-DES patients received ATG. Significantly more non-DES patients showed CMV (27% versus 16%, *p* < 0.001) and EBV (7% versus 4%, *p* = 0.04) negative serology at transplant. Transplant date, age, race, living donor transplant, HLA match, viral-PCR monitoring follow-up period, and sample number tested for viral-PCR per patient were similar in both groups.

### 3.2. The Immune Cell Number before and after Transplant in DES and Non-DES Patients

**Figure 1:**
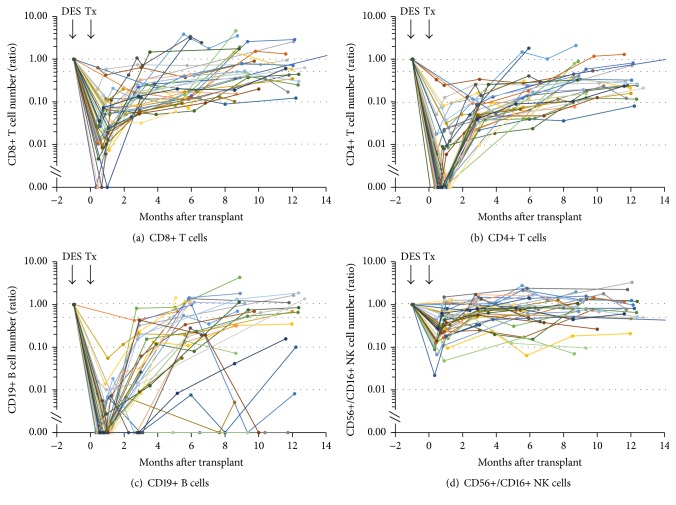
The number of CD8+ (a), CD4+ T cells (b), CD19+ B cells (c), and CD56+/CD16+ NK cells (d) pre-DES and posttransplant in 36 DES patients who received DES with IVIg + rituximab followed by a kidney transplant with alemtuzumab induction. Each line with each symbol describes the result from one patient. The results were expressed as the ratio against the pre-DES level in each patient. DES: desensitization; Tx: transplant.

CD8+, CD4+ T cell, CD19+ B cell, and CD56+/CD16+ NK cell number before DES and after transplant in 36 DES patients are shown in Figure 1. CD19+ B cells were nearly undetectable after rituximab treatment during the DES and continued to be low for several months [39]. For patients receiving alemtuzumab induction after rituximab, the levels at 1 month after transplant (after alemtuzumab) were nearly undetectable (Figure 1(c)). Recovery of CD19+ B cells began 2-3 months after transplant. Although most patients still showed <30% of the pre-DES levels of CD19+ B cells at 6 months after transplant, rapid repopulation was also observed in some patients as previously reported by others [44]. The number of T cells significantly decreased after alemtuzumab induction and restoration began 2-3 months after transplant. It should be noted that the CD4+ T cell numbers at 1 month after transplant were nearly undetectable in most patients (Figure 1(b)), while the CD8+ cell numbers were 1–10% of pre-DES levels (Figure 1(a)). The CD8+ cell number continued to be higher than that of CD4+ cells afterward, which was consistent with our previous report [37]. This trend is likely due to CD4+ T cells being more sensitive than CD8+ T cells to alemtuzumab depletion [45] and/or CD8+ T cell restoration being more rapid than CD4+ T cells [46]. In contrast to B cells and T cells, the reduction of NK cell numbers after transplant was minimal; 20–50% of pre-DES levels were already observed at 1 month after transplant and most patients showed >50% of pre-DES levels by 3 months after transplant (Figure 1(d)). This is consistent with previous observations that NK cells were less susceptible to alemtuzumab depletion [47] and NK cell repopulation was faster than T cells in alemtuzumab-treated cynomolgus monkeys [48].

Alemtuzumab is a monoclonal antibody, targeting CD52 positive cells such as mature lymphocytes, including T cell, B cell, NK cells, and monocytes, and then depleting them [49]. On the other hand, another lymphocyte depleting agent, ATG, is a polyclonal antibody prepared from the sera of rabbits or horses immunized with thymocytes. ATG primarily depletes T cells [19], although induction of B cell apoptosis by ATG was reported [50], resulting in slight reduction of B cells after ATG induction [51]. However, alemtuzumab was reported to be more powerful in reducing T cells than ATG preparations, while reduction of NK cells was similar [51]. In this study, 86% of DES and 49% of non-DES patients received lymphocyte depleting agents, and, of these, 96% of DES patients received alemtuzumab, while 81% of non-DES received ATG. Considering additional B cell depletion by rituximab and alemtuzumab, B cell and T cell depletion in DES was more intense compared to non-DES patients. Theoretically, this should increase the risk for infections in DES patients. Thus, we investigated the viral infection status in DES versus non-DES patients.

### 3.3. Viral Infection in DES and Non-DES Patients

CMV, EBV, and BKV viremia status after transplant in 372 DES and 538 non-DES patients are summarized in Table 2. CMV or EBV DNA levels > 5 copies/PCR and BKV DNA levels > 250 copies/ml as analyzed by our viral-PCR assays were considered viremia, and the levels > 50 copies/PCR and > 2500 copies/ml, respectively, were usually considered for antiviral therapy. Patients with CMV or EBV DNA levels between 30 and 50 copies/PCR and with BKV levels between 1500 and 2500 copies/ml may or may not be treated with antiviral therapy depending on other factors. Thus, the viremia status in the two groups was compared based on 3 viral-PCR cutoff levels. Due to early detection and early intervention, most patients with viremia were asymptomatic.

**Figure 2:**
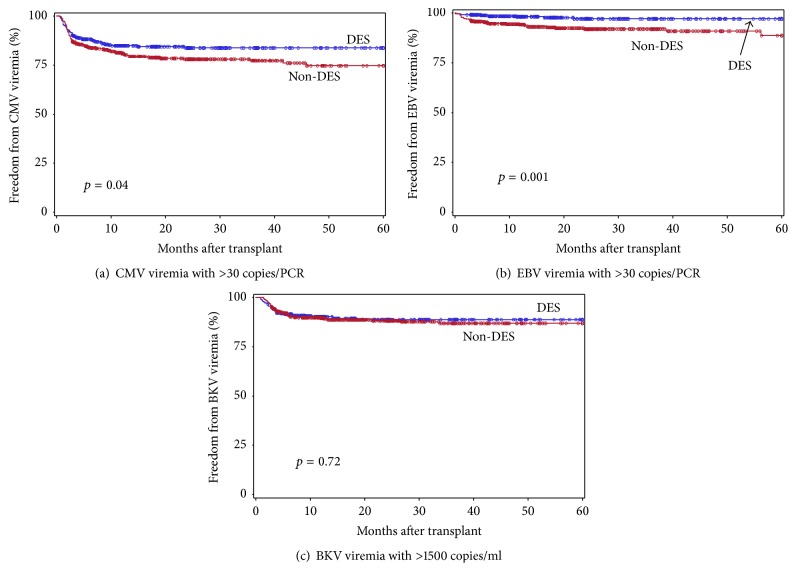
Freedom from CMV (a) or EBV (b) viremia with >30 copies/PCR and BKV (c) viremia with >1500 copies/ml in DES (blue) and non-DES (red) patients during the 1st 5 years after transplant. The group differences were assessed by the log-rank test.

Freedom from CMV or EBV viremia with >30 copies/PCR and BKV viremia with >1500 copies/ml in the DES versus non-DES groups is shown in Figure 2, and the CMV, EBV, or BKV viremia rates with 3 cutoff levels are shown in Table 2. One of the most striking findings in this analysis was the significantly lower CMV and EBV viremia rates in the DES group except for CMV viremia with >5 copies/PCR, and there was no difference in the BKV viremia rates (Table 2, Figure 2). Estimated viremia rates at 5 years after transplant were 30% versus 36% (*p* = 0.19), 16% versus 25% (*p* = 0.04), and 14% versus 23% (*p* < 0.05) for CMV viremia with >5, >30 and >50 copies/PCR, respectively; 14% versus 30% (*p* < 0.001), 2.9% versus 11% (*p* = 0.001), and 2.3% versus 6.4% (*p* = 0.01) for EBV viremia with >5, >30, and >50 copies/PCR, respectively. Significantly shorter duration of CMV viremia (mean months 0.7 versus 1.1 [*p* = 0.02], 0.8 versus 1.4 [*p* = 0.01], and 0.8 versus 1.5 [*p* = 0.01] for viremia with >5, >30, and >50 copies/PCR, resp.) and the trend of lower CMV DNA peak levels were also observed in the DES compared to non-DES group. The 1st CMV viremia with >30 and >50 copies/PCR occurred significantly earlier after transplant in the DES group (mean months after transplant 3.7 versus 6.7 [*p* = 0.02] and 3.4 versus 7.1 [*p* = 0.01], resp.). Viral-PCR monitoring was performed every month during the 1st 3 months after transplant and every 3 months afterwards up to 12 months followed by every 6 months during the 2nd transplant year. Shorter duration of CMV viremia and the trend of lower CMV DNA peak levels observed in DES patients could be due to earlier recognition and treatment in the DES group. No PTLD was seen in either group. There was no significant difference in the BKAN rate or the time to BKAN development in the two groups.

We next analyzed viral infection status separately by pretransplant recipient's viral serology status that largely affects posttransplant viral infection rate and its associated complication [52]. Since pretransplant CMV and EBV sero negativity were significantly higher in the non-DES group, this may have contributed to higher CMV and EBV viremia rates in non-DES patients. We divided DES and non-DES patients into 2 subgroups, CMV sero(+) or (−) and EBV sero(+) or (−). The CMV or EBV viremia status was compared among sero(+), sero(−) DES, sero(+), and sero(−) non-DES patients. The analysis for BKV infection was not performed since BKV serology results were not readily available.

**Figure 3:**
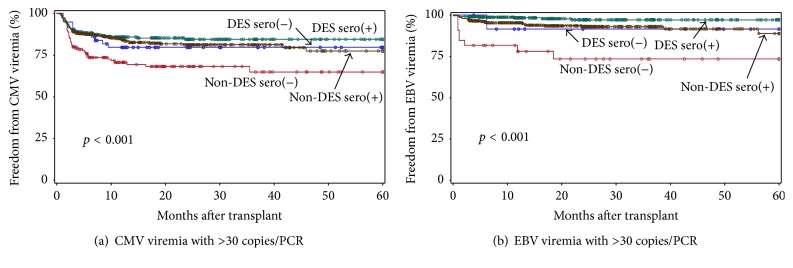
Freedom from CMV (a) or EBV (b) viremia with >30 copies/PCR in sero(+) (green) or sero(−) (blue) DES and sero(+) (brown) or sero(−) (red) non-DES patients during the 1st 5 years after transplant. The group differences were assessed by the log-rank test.

Freedom from CMV or EBV viremia with >30 copies/PCR in the 4 groups is shown in Figure 3, and the CMV or EBV viremia rates with 3 cutoff levels are shown in Tables 3 and 4, respectively. Overall, sero(−) non-DES patients showed least freedom from CMV and EBV viremia during the 1st 5 years after transplant (Figure 3, Tables 3 and 4). In the non-DES group, the CMV and EBV viremia rates were significantly higher in sero(−) versus sero(+) patients (44% versus 33% [*p* = 0.01], 35% versus 23% [*p* < 0.001], and 35% versus 19% [*p* < 0.001] for CMV viremia; 37% versus 32% [*p* = 0.02], 27% versus 11% [*p* < 0.001], and 23% versus 5% [*p* < 0.001] for EBV viremia with >5, >30, and >50 copies/PCR, resp.) (Tables 3 and 4, Figure 3). In contrast, significant difference in CMV and EBV viremia rate between sero(−) versus (+) was not observed in the DES group. When the results were compared among CMV sero(−) patients, the CMV viremia rate was lower in the DES group (22% versus 44% [*p* = 0.03], 20% versus 35% [*p* = 0.09], and 19% versus 35% [*p* = 0.05] for CMV viremia with >5, >30, and >50 copies/PCR, resp.), while the viremia rate was similar in sero(+) DES and non-DES patients (Table 3), suggesting that lower CMV viremia rates observed in DES patients resulted in part from lower CMV viremia in sero(−) DES patients. Among the CMV sero(+) patients, significantly shorter duration of CMV viremia with >30 and >50 copies/PCR was again observed in DES than non-DES patients (mean months 0.7 versus 1.1 [*p* = 0.02], 0.8 versus 1.2 [*p* = 0.03], resp.). This might be due in part to earlier onset of CMV viremia in DES than non-DES patients (mean months after transplant 3.5 versus 8.1, [*p* = 0.02], 3.1 versus 9.2 [*p* = 0.01], resp.). Among EBV sero(+) patients, the EBV viremia rate was significantly or near significantly lower in the DES group: 14% versus 32% [*p* < 0.001], 2.8 versus 11% [*p* < 0.01], and 2.5 versus 5.4% [*p* = 0.09] for viremia with >5, >30, and >50 copies/PCR, respectively (Table 4). Among EBV sero(−) patients, only 2 of 13 (15%) DES patients had viremia with >5 copies/PCR and none showed viremia with >50 copies/PCR during the study period. In contrast, 11 of 33 (33%) non-DES had viremia with >5 copies/PCR and 7 (21%) showed viremia with >50 copies/PCR, suggesting EBV viremia also tended to be lower in sero(−) DES patients, although this difference was not statistically significant. These results suggest that lower EBV viremia rates observed in DES patients resulted from lower EBV viremia in sero(+) and to a lesser degree in sero(−) DES patients. Taken together, the standard protocol used for DES patients affects primarily CMV sero(−) patients to reduce CMV viremia as well as EBV sero(+) and to a lesser degree sero(−) patients to reduce EBV viremia rates. The treatment did not increase the BKV viremia and BKAN rate in DES patients.

ATG is widely used as an induction and rejection treatment agent in transplant patients, and use of a newer lymphocyte depleting agent, alemtuzumab, is also well established [17, 19]. Use of these agents is essential due to their significant reduction of acute rejection, primarily cell-mediated, especially in high risk HS patients. Although the risk for viral infection is a concern due to severe and prolonged lymphocyte depletion, the viral infection risk reported in studies is inconsistent [17, 19, 22, 53–55]. This must be due to various conditions used in these studies such as type of transplantation, the type and dose of maintenance immunosuppressive drugs, application of rejection treatment drugs, type of viruses, viral serological status of recipient, and donor or viral prophylaxis [19]. Several studies showed similar results to ours. Hanaway et al. [17] reported no difference in CMV, EBV, and BKV infections between alemtuzumab versus ATG or anti-IL-2R induction during the first 3 years after transplant in high and low risk kidney transplant recipients with maintenance using tacrolimus, MMF, and 5-day steroid in a regimen of early steroid withdrawal. No significant difference in CMV and BKV infection in kidney transplant recipients who received alemtuzumab versus ATG or anti-IL-2R induction [22, 56], and no difference in CMV, EBV, and HSV infection in HS patients with alemtuzumab versus ATG induction [57] was shown. In contrast to the above study results in HS patients [17, 57], our study showed significantly lower CMV and EBV infection rates in HS (DES) with alemtuzumab compared to non-DES patients with ATG or anti-IL-2R induction. Our HS patients received DES before transplant, while those included in the previously mentioned studies did not. This difference might contribute to the observed lower incidence of CMV and EBV infection in our DES patients.

### 3.4. Possible Factors Contributing to Lower CMV and EBV Viremia Rates in DES Patients

Despite profound and prolonged B cell and T cell depletion from the standard protocol used for DES patients, pretransplant DES with IVIg + rituximab and posttransplant alemtuzumab induction, DES patients showed significantly lower CMV and EBV viremia rates compared to non-DES patients. In the further analysis performed separately by pretransplant CMV and EBV serological status, we found that the standard protocol used for DES patients reduced CMV viremia rate primarily in sero(−) and reduced EBV viremia rate in both sero(+) and sero(−) DES patients to a lesser degree. There are possible factors contributing to these observed beneficial effects of the standard protocol used for DES patients. Possible factors are summarized below.

#### 3.4.1. Viral-Specific T Cells

Viral infections are controlled primarily by antiviral T cells [58]. We have previously shown that CMV-specific CD8+ T cells (CMV-Tc) as analyzed by CFC were detected in most CMV sero(+) healthy individuals as well as kidney transplant recipients, and clearance of CMV DNA was associated with detection of CMV-Tc in those patients [40]. Similar results have also been reported using CFC in solid organ transplant patients [59], ELISPOT in kidney transplant [60], QuantiFeron-CMV® [61], and Tetramer-based assays [62] in allogeneic stem cell transplantation. We also reported that CMV-Tc activity was detected by 2 and 4 months after transplant in 5 of 7 (71%) and 7 of 7 (100%) CMV sero(+) HS patients desensitized with IVIg + rituximab followed by a kidney transplant with alemtuzumab, respectively [39]. Our most recent study in a larger cohort of this patient population (30 patients) also showed similar results [37]: 70% of CMV sero(+) patients showed negativity for CMV-Tc and CMV-specific CD4+ (CMV-Th) cells at 1 month after transplant (after alemtuzumab) due to T cell depletion. However, by 2 months after transplant, 75% showed CMV-Tc and Th cell (+) and 95% did so by 3 months after transplant. These results suggest that a few viral-specific memory T cells that remained after alemtuzumab cell depletion are capable of responding to the virus, resulting in IFN*γ* production and cytotoxic effector functions against infected cells in CMV sero(+) patients. Preservation of memory T cell function following aggressive depletion by alemtuzumab [63, 64] and low risk of virus infection in transplant recipients treated with alemtuzumab [65, 66] have also been reported by other investigators. In the above study [37], we have also shown that one CMV sero(−) patient who developed CMV viremia with >1000 copies/PCR at 2 months after transplant rapidly developed both CMV-Tc and Th, and the viremia was cleared within a month, demonstrating that even CMV sero(−) patients can develop de novo proliferating CMV-T cells after lymphocyte depletion with alemtuzumab. EBV-specific T cells as assessed by IFN*γ* or TNF*α* positivity using the CFC assay were also detected in most EBV sero(+) normal individuals (Figure 5) and transplant recipients (data not shown). Clinical utility of EBV-specific T cell detection in lung, liver, and kidney transplant recipients using tetramer or ELISPOT assays have been reported by other investigators [67, 68]. Taken together, availability of viral-specific T cells in sero(+) patients from early posttransplant and capability of efficiently developing viral-specific T cells in sero(−) patients, despite severe T cell depletion by alemtuzumab, must contribute at least in part to lower CMV and EBV viral infection in this patient population.

#### 3.4.2. Antiviral Antibody

Antiviral antibody functions as one of the early defense mechanisms against viral infection in sero(+) individuals through neutralizing viruses and eliminating virus-infected cells [69–71]. It has been reported that low anti-CMV titer before transplant or at 1 month after transplant was associated with a higher risk of CMV disease in heart transplant recipients [72]. Elevated risk for CMV infection during the 1st year posttransplant was also reported in solid organ transplant recipients with severe hypogammaglobulinemia [73]. In addition, we and others have reported on the benefit of CMV immunoglobulin or IVIg use in the prevention and treatment of viral complications of transplantation including CMV [40, 69, 74], EBV/PTLD [75], parvovirus B19 [76], and BKV infections [21, 77]. These study results demonstrate an important role of antiviral antibody in antiviral immunity in transplant recipients although neutralizing antibodies may not prevent subsequent rounds of infection and the cellular immune response eventually evolves to eradicate the infection [78]. Thus, long-term B cell depletion is always a concern in patients treated with rituximab followed by alemtuzumab induction. Our DES patients often received additional doses of rituximab posttransplant for treatment of ABMR, which may result in more prolonged B cell depletion and possible reduction of antibodies including antiviral antibodies.

We have previously reported [39] that total IgG, IgM, and IgA levels significantly decreased 4–10 months after DES (equivalent to average 9.6 months after transplant) compared to the pre-DES levels in 14 HS kidney transplant recipients with alemtuzumab induction. However, the reduction was only 15–20% and the reduced levels were still within the normal range in most patients. Minimal changes or moderate reductions (15–20% reduction) in total Ig during 6–12 months after rituximab treatment have also been shown in patients with arthritis [79], those with relapsing-remitting multiple sclerosis [80], and those with active rheumatoid arthritis [81]. In addition, anti-CMV IgG levels in CMV sero(+) patients did not change from pre-DES levels up to 10 months after transplant. Based on these results, we suggested that anti-CMV IgG might be produced primarily by CD20− long-lived plasma cells that are not affected by rituximab [82], while 15–20% of total IgG and IgM and IgA producing B cells might be CD20+ peripheral B cells and/or CD20− short-lived plasma cells [83].

**Figure 4:**
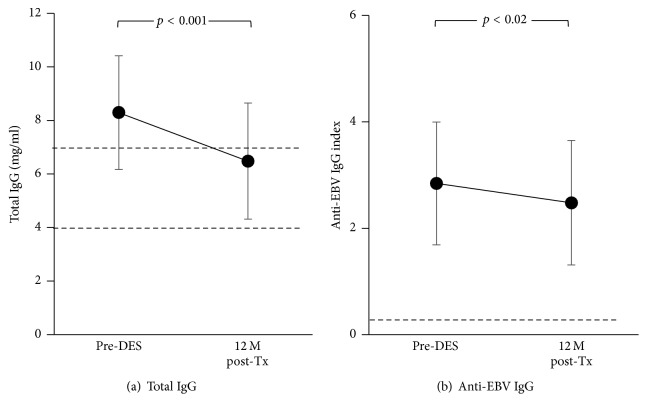
Total IgG (a) and anti-EBV IgG levels (b) before DES and at 12 months (M) after transplant (Tx) in 35 and 33 DES patients, respectively, who received DES with IVIg + rituximab followed by a kidney transplant with alemtuzumab induction. The results are expressed as mean and standard deviation. The dotted lines describe 7 and 4 mg/ml total IgG for the normal and severe hypogammaglobulinemia cutoff, respectively, in (a), and anti-EBV IgG index 0.25 for sero(+) cutoff level in (b).

In this study, we measured total IgG and anti-EBV IgG levels before DES and 12 months after transplant (15.7 ± 2.9 months after DES) in 35 patients who received DES with IVIg + rituximab, followed by a kidney transplant with alemtuzumab induction. Total IgG levels significantly decreased at 12 months after transplant compared to pre-DES levels (22% reduction) (Figure 4(a)), which is consistent with our previous results [39]. However, mean total IgG levels at 12 months after transplant was 6.5 mg/ml that is close to normal level, >7 mg/ml, and all patients except for two showed levels >4 mg/ml. Levels <4 mg/ml, considered severe hypogammaglobulinemia, are often associated with increased risk of viral and fungal infections and higher mortality [73, 74]. Pre-DES levels in the two patients with posttransplant total IgG <4 mg/ml were 2.5 and 6.2 mg/ml, already lower than normal levels. Florescu et al. reported based on a meta-analysis [73] that 45% of solid organ transplant recipients had hypogammaglobulinemia (total IgG < 7 mg/ml) within the 1st year after transplant and 15% had severe hypogammaglobulinemia (<4 mg/ml). In the current study, 20/35 patients (57%) showed total IgG <7 mg/ml that was slightly higher than their report, but only 2/35 (6%) showed severe hypogammaglobulinemia requiring transient treatment with IVIg.

In contrast to posttransplant anti-CMV IgG levels observed in the previous study [39], anti-EBV IgG levels significantly decreased in EBV sero(+) patients at 12 months after transplant in this study (Figure 4(b)). However, the reduction was minimal, 11%. Taken together, consistently available anti-CMV and anti-EBV IgG that are not affected by prolonged B cell depletion must contribute at least in part to lower CMV and EBV viral infection in sero(+) DES patients.

#### 3.4.3. Antibody-Dependent Cell-Mediated Cytotoxicity (ADCC)

ADCC is one of the major antiviral activities and mediated by Fc*γ*RIIIa (CD16) bearing cells such as NK cells, monocytes, and a subset of CD8+ T cells through interaction of CD16 with Fc portion of antiviral IgG bound to the viral infected targets [70, 71, 84, 85]. Among the CD16 bearing cells, NK cells are primarily responsible for ADCC. As shown in Figure 1 and in previous studies [37, 39], CD56+/CD16+ NK cell numbers did not decrease as much as T cells after alemtuzumab induction in DES patients. In this study, 20–50% of pre-DES NK cell levels were already detected at 1 month after transplant and >50% of pre-DES levels by 3 months (Figure 1(d)) in most patients. In addition, anti-CMV and anti-EBV IgG levels showed no or minimal changes after transplant (after alemtuzumab) in CMV and EBV sero(+) patients, respectively [39] (Figure 4). These results suggest that NK cells-anti-CMV or anti-EBV IgG-mediated ADCC may be another factor contributing to lower rate of CMV or EBV infection in sero(+) DES patients.

**Figure 5:**
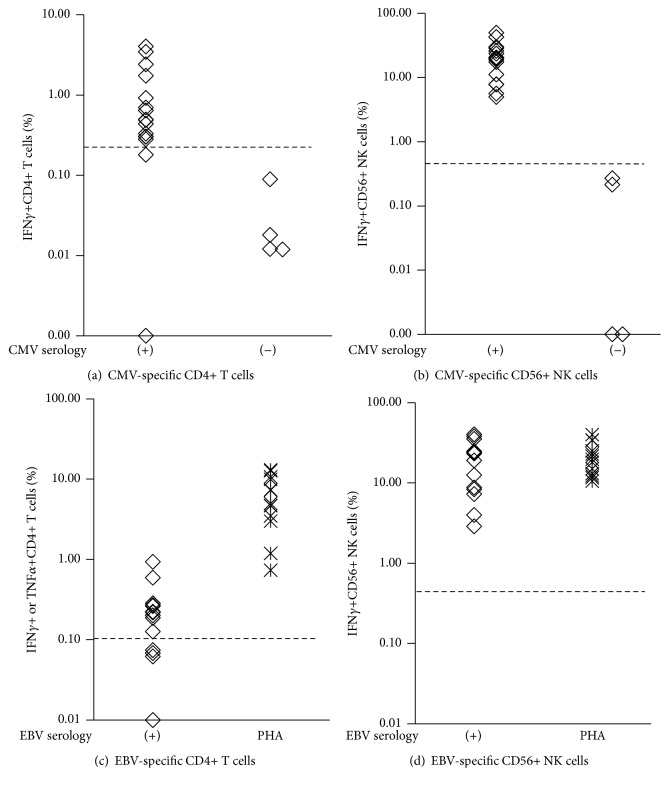
The levels of CMV-specific CD4+ T cells (CMV-Th) (a) and CD56+ NK cells (CMV-NK) (b) in 16 CMV sero(+) and 4 sero(−) normal individuals and EBV-specific CD4+ T cells (EBV-Th) (c) and CD56+ NK cells (EBV-NK) (d) in 14 EBV sero(+) normal individuals as analyzed by CFC. Each symbol represents the result from one individual. The dotted line describes the positive cutoff level; ≥0.2% for CMV-Th, ≥0.1% for EBV-Th, and ≥0.5% for CMV- and EBV-NK. The PHA (+) control results were also shown in (c) and (d).

To address this possible ADCC activity in sero(+) patients, we measured NK cell response to CMV or EBV lysate (CMV- or EBV-NK) in vitro by assessing IFN*γ* production and CD107a expression, degranulation marker [41, 86], using a CFC technique. We first measured the IFN*γ*+ cell% in CD4+ T and CD56+ NK cells in response to CMV in 20 and EBV lysate in 14 normal individuals; 4 of 20 were CMV sero(−) and all 14 were EBV sero(+). Most CMV sero(+) individuals except for two showed CMV-Th(+) (≥0.2%), while all 4 sero(−) individuals were (−) for CMV-Th (Figure 5(a)). Similar responses were seen in all 16 CMV sero(+) individuals showing (+) for CMV-NK (≥0.5%), while all sero(−) showed CMV-NK(−) (Figure 5(b)). In the EBV-Th and NK assay, 10 of 14 showed EBV-Th(+) (≥0.1%) and the remaining 3 were (−) (Figure 5(c)), while all 14 showed (+) for EBV-NK (Figure 5(d)). All PHA positive controls were (+) for EBV-Th and NK (Figures 5(c) and 5(d)).

**Figure 6:**
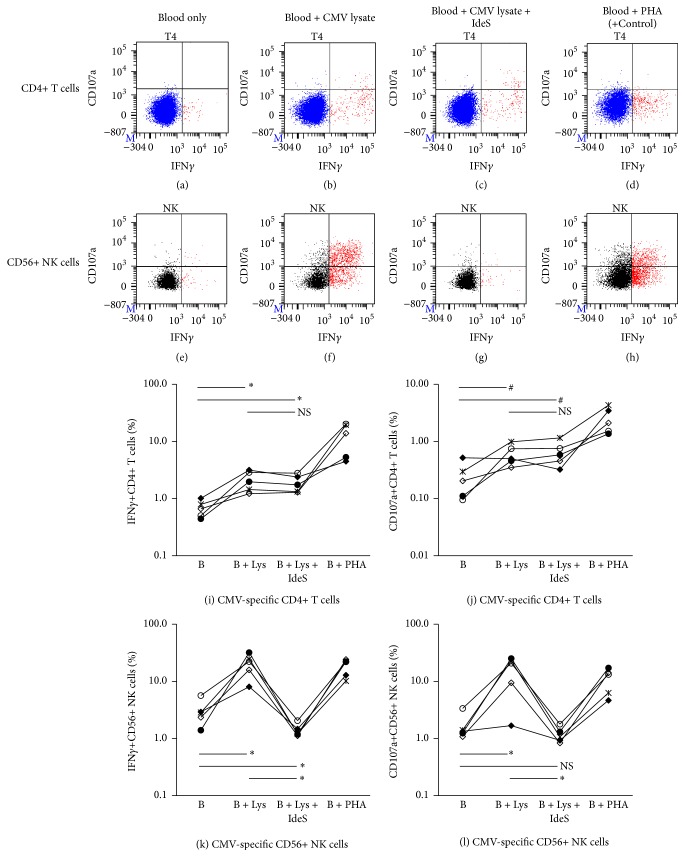
Involvement of anti-CMV IgG in positive reactivity of CMV-Th and CMV-NK cells. Upper (a–h): a typical result of the CMV-Th and CMV-NK assay performed with or without IdeS, IgG-degrading enzyme. Lower (i–l): the summary of 5 sets of experiment results using 5 different normal individuals. Each line with each symbol describes the result from one normal individual. B: blood; Lys: CMV lysate. ^*∗*^*p* < 0.05, ^#^*p* = 0.05–0.1, and NS: not significant (*p* ≥ 0.1) as assessed by paired *t*-test.

**Figure 7:**
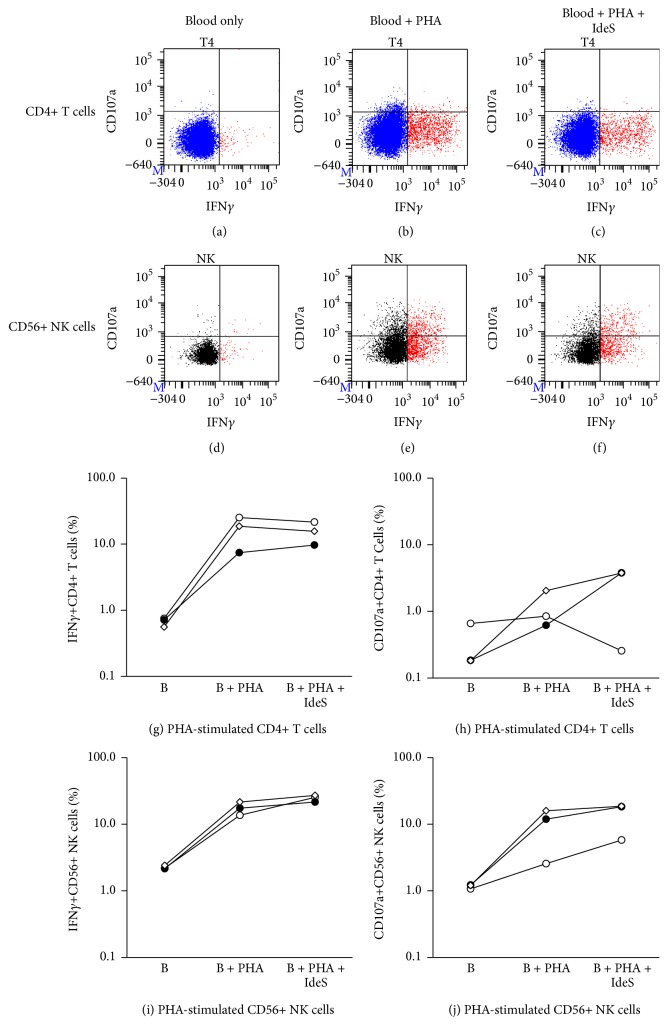
The effect of IdeS on CD4+ T cell and CD56+ NK cell activation in response to PHA. Upper (a–f): a typical result of CD4+ T cell and CD56+ NK cell response to PHA (positive control) with or without IdeS in the CMV-Th and CMV-NK assay. Lower (g–j): the summary of 3 sets of experiment results using 3 different normal individuals. Each line with each symbol describes the result from one normal individual. B: blood.

It is well accepted that CMV- or EBV-T cells are viral-specific memory T cells and their response to CMV or EBV peptides or lysate in vitro are mediated through T cell receptors [40, 59, 67, 68]. NK cells express various receptors that are critical to their function and have traditionally been classified as important effectors of the innate immune system [87]. As characterization of NK cells has advanced, their crucial role in immunity has been reaffirmed and expanded [88, 89]. Recent studies suggest that NK cells have the capacity for immunological memory [90, 91]. To determine if the NK cell activation in response to CMV lysate observed in this study was mediated by direct interaction of NK cell receptors with CMV antigens on the lysate or antigen presenting cells, or an indirect interaction of NK cells with CMV antigens via CD16 and anti-CMV antibody interaction, similar experiments were performed using IdeS, an IgG-degrading enzyme [92], which enzymatically digest intact IgG into F(ab′)_2_ and Fc fragments, in 5 CMV sero(+) normal individuals, with subsequent analysis of the IFN*γ*+ cell% and CD107a+ cell% in CD4+ T and CD56+ NK cells. IFN*γ*+ and CD107a+ cell% in CD4+ T cells and CD56+ NK cells were elevated when blood was stimulated with CMV lysate (Figures 6(b), 6(f), and 6(i)–6(l)). The elevated IFN*γ*+ and CD107a+ cell% in CD4+ T cells remained when blood pretreated with IdeS was used for the assay (Figures 6(c), 6(i), and 6(j)). In contrast, the elevated IFN*γ*+ and CD107a+ cell% in CD56+ NK cells was completely abrogated when blood was pretreated with IdeS (Figures 6(g), 6(k), and 6(l)). These results suggest that CD4+ T cell activation in response to CMV lysate is anti-CMV antibody-independent and is memory T cell response specific to CMV antigens via T cell receptor as expected, while CD56+ NK cell activation in response to CMV lysate is anti-CMV antibody-dependent via CD16 and the involvement of other NK cell receptors is unlikely as NK cell activation was similar to background level after IdeS treatment. To determine if the complete inhibition of IFN*γ* production and CD107a expression by IdeS in NK cells was due to an interaction of IdeS with NK cells, blood stimulated with PHA (positive control) was incubated with or without IdeS in the CMV-T and NK assay. As shown in Figure 7, the elevated IFN*γ*+ and CD107a+ cell% in NK cells stimulated with PHA remained elevated even when blood was treated with IdeS (Figures 7(e), 7(f), 7(i), and 7(j)), suggesting that IdeS has no direct suppressive effect on NK cells. The same is true for the elevated IFN*γ*+ cell% in CD4+ T cells (Figures 7(b), 7(c), and 7(g)), while CD107a expression in PHA-stimulated CD4+ cells was minimal (Figures 7(b), 7(c), and 7(h)). Taken together, these results demonstrate that it is likely that ADCC plays an important role in prevention and reduction of CMV and EBV infection in sero(+) DES patients, especially during the first 1-2 months after transplant when less antiviral T cells are available in many patients due to alemtuzumab induction. ADCC-mediated antiviral activity is also likely to take a part of controlling viral infection in sero(−) patients who are treated with CMV-Ig or IVIg as it has been reported that elimination of CMV or EBV-infected cells by CMV-Ig or IVIg was enhanced by addition of NK cells via ADCC [85].

Recent studies showing the important role of antibody-mediated NK cell activity beyond a traditional ADCC mechanism in controlling CMV infection are of interest [93–95]. Elevated number and activity of NK cells during CMV infection in transplant recipients were previously reported [94]. Recently, NKG2C^hi^CD57^hi^ NK cells have been identified to be expanded exclusively at CMV infection [93, 96, 97] and its effector function is enhanced only in the presence of anti-CMV antibodies [95]. IFN*γ*+ and CD107a+ NK cells activated via antibody in response to CMV lysate as detected in the CMV-NK assay of this study might be this particular NK cell subset.

#### 3.4.4. Lymphocyte Depletion

Contrary to the widely held concept that lymphocyte depletion increases risk for viral infections, several studies including a meta-analysis showed a similar viral infection rate among patients treated with alemtuzumab, ATG, and anti-IL-2R induction [17, 22, 56]. Our DES patients included in this study received an additional lymphocyte depleting agent, rituximab, for pretransplant DES and treatment of ABMR in some patients, and these patients showed even lower CMV and EBV infection rate compared to non-DES patients (Tables 2, 3, and 4 and Figures 2, 3), suggesting that lymphocyte depletion itself might be one of possible factors for the lower viral infection rate in this patient population.

EBV enters B cells via the C3d complement receptor CD21 and establishes its latency on B cells [98]. As EBV is detected in a wide range of B cells, from resting B cells through blast cells to fully differentiated plasma cells, B cells are considered the primary reservoir for EBV [99]. Our study showed that the EBV viremia rates were significantly lower in EBV sero(+) DES and also tended to be lower in EBV sero(−) DES patients (Table 4). Elimination of EBV reservoirs, B cells, by rituximab and alemtuzumab used for DES and induction therapy may reduce the reactivation of latent EBV and/or primary infection in this patient population. In fact, only 2/13 (15%) EBV sero(−) DES patients developed EBV viremia, one with <50 and another with <30 copies/PCR, and none had EBV viremia with >50 copies/PCR, which was in contrast to 7/33 (21%) in EBV sero(−) non-DES patients, although this was not statistically significant (*p* = 0.08, Table 4). Earlier studies showed no EBV reactivation in patients previously treated with rituximab [100, 101] and an association of a lower risk for PTLD by treatment with T cell and B cell depleting agent, alemtuzumab, compared to an agent depleting only T cells, ATG, in hematopoietic cell transplantation [29]. Recently, Schachtner and Reinke [102] reported that a single dose of rituximab 4 weeks prior to transplant significantly reduced posttransplant EBV viremia in EBV sero(−) kidney transplant recipients who received EBV sero(+) kidney, compared to those without rituximab treatment, and no patient developed PTLD. These patients received induction with either anti-IL-2R or ATG, and maintenance with calcineurin inhibitor + MMF + steroid. Thus, we think that the low incidence of EBV viremia observed in DES patients of our study is likely due to rituximab used for DES, although a subsequent use of alemtuzumab induction might further increase this beneficial effect in DES patients.

After primary CMV infection, the virus can persist in a latent form in a variety of tissues, primarily in monocyte-derived macrophages and dendritic cells [29, 103, 104]. The CMV viremia rate was reduced in DES compared to non-DES patients, primarily in CMV sero(−) DES patients. (Table 3). Monocytes and myeloid dendritic cells express CD52 at high levels, but these are less susceptible to alemtuzumab-mediated complement-dependent cell cytotoxicity due to high levels of complement inhibitory proteins expressed on these cells [47]. Thus, unlike B cells for EBV, it is unlikely that the elimination of CMV reservoir is a major reason for lower CMV viremia rate observed in DES patients.

BKV establishes latency in the uroepithelium after a primary infection, [6, 105]. Reactivation and replication occur in immunocompromised patients such as transplant recipients, resulting in viruria. When replication is aggressive, BKV viremia emerges due to injured renal epithelial cells. Currently, it is well accepted that the primary reservoir for BKV is renal epithelial cells, and suggested that the source of BKV in plasma is derived from BKV replication in the allograft of kidney transplant recipients [105]. Thus, alemtuzumab and rituximab have no ability to reduce or prevent BKV infection through elimination of BKV reservoir.

#### 3.4.5. Antiviral Prophylaxis

Antiviral prophylaxis or preemptive antiviral therapy is essential for prevention of viral infections and associated complications in transplant recipients [106]. Both strategies are acceptable, but differences are noted [107–110]. Currently, ganciclovir and valganciclovir are commonly used as first-line antiviral prophylaxis and are most effective in preventing CMV infection and disease, although inhibition of other herpes viruses (herpes simplex virus types 1 and 2 [HSV-1, HSV-2], EBV, varicella-zoster virus [VZV], and human herpes virus 6 [HHV-6]) is noted [106, 110]. Acyclovir is also used as antiprophylaxis agent but does not have significant efficacy against CMV compared to HSV-1 and HSV-2, VZV, and EBV [108, 110].

All patients included in this study received antiviral prophylaxis with ganciclovir while inpatient and then valganciclovir or acyclovir posttransplant depending on a risk for CMV infection based on donor and recipient CMV serologies. Briefly, for transplants with lymphocyte depletion induction and those with CMV R−/D+ regardless of induction type, valganciclovir was given. For those with anti-IL-2R induction and CMV R+/D+, R+/D−, or R−/D−, acyclovir was given. In this study, we showed that CMV and EBV viremia rate in DES patients were significantly lower than non-DES patients. Most DES patients (86%) received ganciclovir since they received lymphocyte depletion induction, while 51% of the non-DES patients received acyclovir due to anti-IL-2R induction and CMV sero-status. This difference in antiviral prophylaxis may be a reason for differences in viremia rates in the DES versus non-DES groups. To address this question, we compared the CMV and EBV viremia rates in non-DES patients with valganciclovir versus acyclovir. Since viral sero-status affects the viremia rate, only CMV or EBV sero(+) non-DES patients were included in this analysis. Among CMV sero(+) non-DES patients, 176 received valganciclovir and 175 received acyclovir (Table 5). No significant difference in CMV viremia with >30 copies/PCR was found in the two groups. Among EBV sero(+) non-DES patients, even those with valganciclovir showed higher EBV viremia rates compared to those with acyclovir (22 of 200 [11%] versus 6/215 [2.8%], *p* < 0.001). These results indicate that higher CMV and EBV viremia rates observed in non-DES versus DES patients are unlikely due to acyclovir given to more non-DES patients. Since the most common induction agent used for DES patients was alemtuzumab and most non-DES patients received ATG, we further analyzed the viremia rate in sero(+) non-DES patients with valganciclovir who received alemtuzumab versus ATG. No significant difference in the CMV or EBV viremia rate was found (Table 5), suggesting that the difference in lymphocyte depleting agents is unlikely the reason for higher CMV and EBV viremia rates in non-DES patients in our patient population. It should be noted that non-DES patients who received alemtuzumab showed less EBV viremia rate compared to those with ATG (5% versus 12%) as mentioned in the previous section, but this was not statistically significant.

#### 3.4.6. IVIg

IVIg derived from pooled human plasma from thousands of donors and originally used for the treatment of primary immunodeficiency disorders has also been used for the treatment of autoimmune and inflammatory disorders for nearly 30 years [74, 111, 112] and is currently recognized as a potent immunomodulatory agent. It affects innate and adaptive immune systems, and its effect on most components of immune system including antibodies, complements, cytokines, most immune cells and their receptors, and the interaction of these components have been reported [74, 111, 112]. Precise mechanisms of immune modulation are still not well known although various possible mechanisms have been proposed depending on diseases or its clinical application. We have been using IVIg for the DES therapy as an immunomodulatory agent against allosensitization as previously reported [74, 113–116]. Antiviral properties found in IVIg as discussed at earlier section became an additional benefit for our DES patients. The low CMV and EBV viremia rate observed in DES compared to non-DES patients might be due in part to this IVIg effect.

### 3.5. Impact of Viral Infection on Allograft Rejection

**Figure 8:**
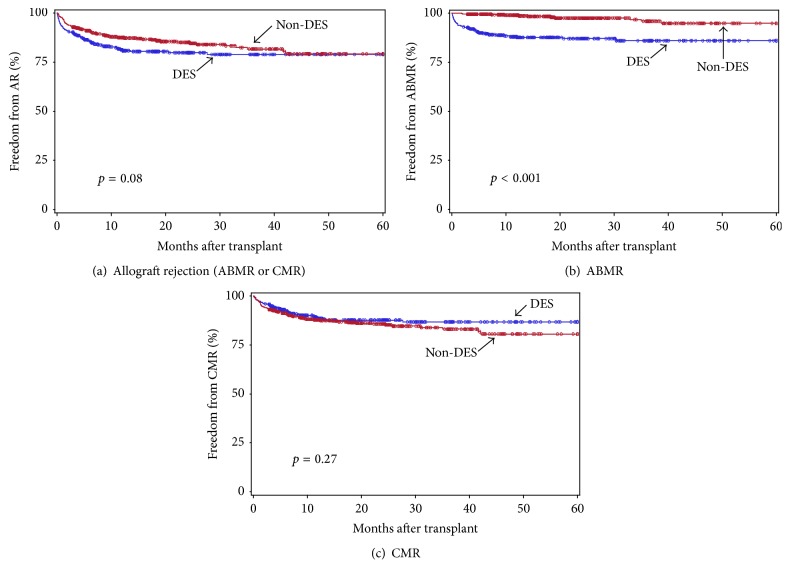
Freedom from overall allograft rejection (a), ABMR (b), and CMR (c) in DES (blue) versus non-DES (red) patients during the 1st 5 years after transplant. The group differences were assessed by the log-rank test.

We next investigated the impact of viral infection on allograft rejection. Of 372 DES and 538 non-DES patients, the rejection information was available in 363 DES and 497 non-DES patients during this study period. Freedom from total allograft rejection, ABMR, and CMR in DES versus non-DES patients is shown in Figure 8. The DES group showed less freedom from overall allograft rejection (*p* = 0.08, Figure 8(a)) during the 1st 5 years after transplant, but this was not statistically significant. When the analysis was performed separately by ABMR and CMR, freedom from ABMR in the DES group was significantly less than non-DES group (*p* < 0.001, Figure 8(b)), which is common and a major obstacle in HS patients [12–15], while rates of CMR were similar in the two groups (*p* = 0.27, Figure 8(c)). This demonstrated that the trend of less freedom from overall rejection observed in the DES was due to significantly less freedom from ABMR in the DES group. The number of patients who had overall allograft rejection (19% versus 14%, resp.), ABMR (12% versus 2.2%), or CMR (11% versus 14%) during this study period in the DES and non-DES groups is also shown in Table 6.

To assess the impact of viral infection on induction of allograft rejection, we next analyzed the rejection rate within 6 months after onset of viral infection. Of 363 DES and 497 non-DES patients, 80 (22%) and 138 (28%) developed at least one CMV with >50, EBV with >50 copies/PCR, or BKV viremia with >2500 copies/ml during this study period (Table 6). Of these, 19% in the DES and 12% in the non-DES groups developed either ABMR or CMR within 6 months after viral infection, which was similar to the overall rejection rate in the whole DES and non-DES groups as shown above (19% versus 14%). In addition, the AMR rate within 6 months after infection in the DES and non-DES groups (10% and 2.2%, resp.) is again similar to that in the whole DES and non-DES groups (12% versus 2.2%), and the same is true for the CMR rate after infection (14% versus 11%). These results suggest that the impact of viral infection on induction of allograft rejection occurring within 6 months after infection is minimal in our patients, both DES and non-DES patients.

The increased risk of viral infections and their complications associated with antirejection therapy is well documented [28, 117]. On the other hand, acute and chronic allograft injuries and rejections caused directly and indirectly by viral infections have also been suggested, but not conclusive [28, 29]. In kidney transplant, CMV infection is known to mediate allograft injury and rejection likely through systemic inflammation, cytokines, and T cell activation induced by CMV [28, 117, 118]. However this trend has been dramatically reduced after application of anti-CMV prophylaxis [28, 107, 119]. All our patients, DES and non-DES, received antiviral prophylaxis for 6 months after transplant, which may minimize the impact of CMV infection on induction of allograft rejection.

### 3.6. Allograft and Patient Survival in DES and Non-DES Patients

**Figure 9:**
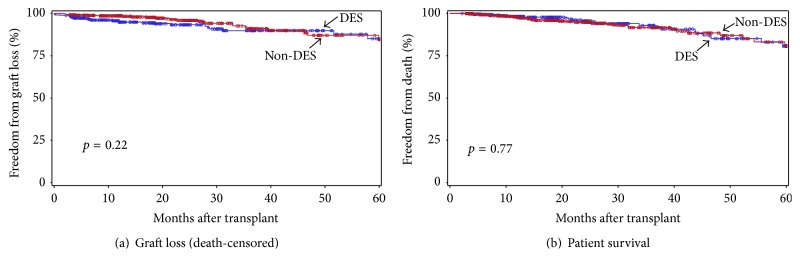
Freedom from allograft loss (a) and patient death (b) in DES (blue) versus non-DES (red) patients during the 1st 5 years after transplant. The group differences were assessed by the log-rank test.

We finally compared allograft and patient survival in DES versus non-DES patients included in this study (Figure 9). There was no significant difference in both allograft and patient survival in the two groups, which was previously reported in our other studies [14, 15, 26, 27]. The estimated graft (death-censored) and patient survival at 2 years after transplant were 93.4% and 95.6% in the DES and 96.1% and 94.9% in the non-DES groups, respectively.

## 4. Conclusions

Desensitized HS patients are at lower risk for CMV and EBV infections and have a similar risk for BKV infection and BKAN posttransplant. This trend was observed primarily in CMV sero(−) for CMV infection and in EBV sero(+) and sero(−) patients to a lesser degree for EBV infection. No patient developed PTLD in either group. Factors likely responsible for the lower risks for CMV and EBV infections in DES patients include (1) viral-specific memory T cells remaining after lymphocyte depletion with alemtuzumab are capable of efficiently proliferating to clear virus in sero(+) patients. In addition, sero(−) patients are capable of efficiently developing viral-specific T cells even after T cell and B cell depletion; (2) high levels of NK cells remaining after alemtuzumab and consistently available antiviral IgG after T cell and B cell depletion contribute to clearance of CMV and EBV through ADCC in sero(+) patients; (3) elimination of B cells (EBV reservoirs) by rituximab and alemtuzumab, and to a lesser degree, monocytes (CMV reservoir) by alemtuzumab, may reduce or prevent the reactivation of latent infection and/or primary infection; (4) the use of IVIg which contains potent antiviral IgGs that likely have a beneficial effect in preventing or modulating viral infections. We have recently reported that B cell and T cell depletion is unlikely to increase a risk for polyomavirus JC (JCV) and progressive multifocal leukoencephalopathy (PML) in DES patients [24]. Allograft and patient survival were similar in both groups. Taken together, we conclude that the IVIg + rituximab DES combined with alemtuzumab induction with triple immunosuppression maintenance does not increase risk for CMV, EBV, BKV, and JCV infections and their associated complications including PTLD, BKAN, and PML in HS kidney transplant patients under antiviral surveillance with antiviral prophylaxis for 6 months after transplant and close monitoring viral infection by PCR for early detection and early intervention.

## Figures and Tables

**Table 1:** Patient demographics.

Demographics	Kidney transplant patients		*p* value
	DES group (*n* = 372)	Non-DES group (*n* = 538)	
Transplant date	1/4/07–4/18/15	1/10/07–4/17/15	
Age, mean ± SD	49.6 ± 13.3	51.1 ± 14.2	0.11
Gender (female), *n* (%)	226 (60.8)	157 (29.2)	**<0.001**
Race, *n* (%)			0.27
African-American	69/368 (18.8)	84/499 (16.8)	
Hispanic	101/368 (27.4)	167/499 (33.5)	
White	148/368 (40.2)	180/499 (36.1)	
Others	50/368 (13.6)	68/499 (13.6)	
Living donor transplant, *n* (%)	141 (37.9)	177 (32.9)	0.12
Induction, *n* (%)			**<0.001**
Lymphocyte depletion	312/365 (85.5)	241/496 (48.6)	
Anti-IL-2 receptor	53/365 (14.5)	255/496 (51.4)	
Maintenance (tacrolimus), *n* (%)	351/354 (99.2)	452/493 (91.7)	**<0.001**
HLA match^*∗*^, mean ± SD	1.9 ± 1.6	1.9 ± 1.7	0.78
PRA, *n* (%)			**<0.001**
>10%	66 (17.7)	470 (87.3)	
10–80%	113 (30.4)	68 (12.7)	
>80%	193 (51.9)	0 (0)	
Reason for DES, *n* (%)			
HS	314 (84.4)	na	
HS/ABOi	17 (4.6)	na	
ABOi	41 (11.0)	na	
Recipient with CMV sero(−) at Tx, *n* (%)	57/368 (15.5)	140/524 (26.7)	**<0.001**
Recipient with EBV sero(−) at Tx, *n* (%)	13/361 (3.6)	33/486 (6.8)	**0.04**
Follow-up (months post-Tx)^*∗∗*^, mean ± SD	24.4 ± 20.3	24.6 ± 20.1	0.84
Sample number tested for viral-PCR/patient, mean ± SD	9.7 ± 5.2	9.5 ± 7.4	0.61

^*∗*^HLA match in 350 DES and 392 non-DES patients with available results. ^*∗∗*^Follow-up for viral-PCR monitoring.

The number of patients with available results was provided if not available in all the patients. PRA: panel reactive antibody, DES: desensitization, HS: HLA-sensitization, ABOi: ABO incompatible transplantation, and Tx: transplant.

**Table 2:** CMV, EBV, and BKV viremia in DES and non-DES patients.

Viral DNA detected	Kidney transplant patients		*p* value
	DES group (*n* = 372)	Non-DES group (*n* = 538)	
CMV-PCR > 5.0 copies/PCR			
Viremia rate (% ± SE)^*∗*^	30.3 ± 3.6	35.7 ± 2.9	0.19
1st viremia (m post-Tx)	5.8 ± 10.3	7.7 ± 14.1	0.23
Peak levels (copies/PCR)	420 ± 1077	2730 ± 18374	0.12
Duration (m)	0.7 ± 0.7	1.1 ± 2.0	**0.02**
CMV-PCR > 30 copies/PCR			
Vremia rate (% ± SE)^*∗*^	16.1 ± 2.1	25.2 ± 2.7	**0.04**
1st viremia (m post-Tx)	3.7 ± 4.0	6.7 ± 12.5	**0.02**
Peak levels (copies/PCR)	699 ± 1331	3904 ± 21887	0.13
Duration (m)	0.8 ± 0.5	1.4 ± 2.4	**0.01**
CMV-PCR > 50 copies/PCR			
Viremia rate (% ± SE)^*∗*^	13.5 ± 1.9	22.5 ± 2.7	**<0.05**
1st viremia (m post-Tx)	3.4 ± 3.1	7.1 ± 13.3	**0.01**
Peak levels (copies/PCR)	811 ± 1410	4462 ± 23360	0.13
Duration (m)	0.8 ± 0.6	1.5 ± 2.5	**0.01**

EBV-PCR > 5.0 copies/PCR			
Viremia rate (% ± SE)^*∗*^	13.6 ± 3.8	30.0 ± 4.5	**<0.001**
1st viremia (m post-Tx)	18.8 ± 19.5	13.1 ± 19.3	0.20
Peak levels (copies/PCR)	171 ± 512	74 ± 141	0.36
Duration (m)	6.7 ± 17.5	4.5 ± 6.4	0.54
EBV-PCR > 30 copies/PCR			
Viremia rate (% ± SE)^*∗*^	2.9 ± 1.1	11.3 ± 2.8	**0.001**
1st viremia (m post-Tx)	14.5 ± 20.2	9.4 ± 15.5	0.52
Peak levels (copies/PCR)	474 ± 785	157 ± 187	0.29
Duration (m)	17.1 ± 26.6	7.5 ± 7.7	0.34
EBV-PCR > 50 copies/PCR			
Viremia rate (% ± SE)^*∗*^	2.3 ± 1.0	6.4 ± 1.5	**0.01**
1st viremia (m post-Tx)	9.4 ± 7.8	6.4 ± 8.4	0.47
Peak levels (copies/PCR)	691 ± 884	216 ± 205	0.29
Duration (m)	23.6 ± 30.6	8.3 ± 8.1	0.32
PTLD, *n* (%)	0	0	na

BKV-PCR > 250 copies/ml			
Viremia rate (% ± SE)^*∗*^	20.1 ± 2.5	17.1 ± 1.9	0.21
1st viremia (m post-Tx)	5.2 ± 5.4	6.6 ± 8.7	0.25
Peak levels (copies/PCR)	7.2 × 10^5^ ± 4.3 × 10^6^	1.6 × 10^5^ ± 5.8 × 10^5^	0.31
Duration (m)	5.0 ± 10.4	5.8 ± 11.1	0.70
BKV-PCR >1500 copies/ml			
Viremia rate (% ± SE)^*∗*^	11.2 ± 1.8	13.0 ± 1.8	0.72
1st viremia (m post-Tx)	4.2 ± 4.0	6.4 ± 9.7	0.14
Peak levels (copies/PCR)	1.2 × 10^6^ ± 5.5 × 10^6^	2.1 × 10^5^ ± 6.6 × 10^5^	0.28
Duration (m)	7.8 ± 12.8	7.3 ± 12.2	0.84
BKV-PCR >2500 copies/ml			
Viremia rate (% ± SE)^*∗*^	10.9 ± 1.8	10.7 ± 1.7	0.60
1st viremia (m post-Tx)	4.3 ± 4.1	6.9 ± 10.6	0.12
Peak levels (copies/PCR)	1.3 × 10^6^ ± 5.6 × 10^6^	2.5 × 10^5^ ± 7.2 × 10^5^	0.30
Duration (m)	8.0 ± 13.0	8.4 ± 13.2	0.88
BKAN, *n* (%)	4 (1.1)	10 (1.9)	0.35
Time for BKAN (m post-Tx)	12.3 ± 10.1	11.6 ± 7.1	0.92

^*∗*^The viremia rates (%  ± standard error [SE]) at 5 years after transplant (Tx) were estimated by the Kaplan-Meier method and the group differences were assessed by the log-rank test.

Results for 1st viremia, peak levels, duration, and time for BKAN are mean ± standard deviation.

m post-Tx: months after transplant, PTLD: posttransplant lymphoproliferative disorder, and BKAN: BKV-associated nephropathy.

**Table 3:** CMV viremia in CMV sero(+) versus sero(−) patients in the DES and non-DES groups.

	DES group (*n* = 368)			Non-DES group (*n* = 524)			*p* value (DES versus non-DES)	
	CMV sero(+) (*n* = 311)	CMV sero(−) (*n* = 57)	*p* value (sero+ versus −)	CMV sero(+) (*n* = 384)	CMV sero(−) (*n* = 140)	*p* value (sero+ versus −)	In CMV sero(+)	In CMV sero(−)
CMV-PCR > 5.0 copies/PCR								
Viremia rate (% ± SE)^*∗*^	32.0 ± 4.0	22.1 ± 5.7	0.37	33.4 ± 3.5	43.5 ± 5.5	**0.01**	0.95	**0.03**
1st viremia (m post-Tx)	6.0 ± 11.0	4.3 ± 2.8	0.25	9.1 ± 16.7	4.9 ± 5.9	**0.02**	0.14	0.60
Peak levels (copies/PCR)	237 ± 578	1652 ± 2236	**0.06**	2951 ± 22399	2407 ± 4370	0.81	0.22	0.42
Duration (m)	0.7 ± 0.6	0.9 ± 0.6	0.22	0.9 ± 1.1	1.6 ± 3.1	0.11	0.15	0.17
CMV-PCR > 30 copies/PCR								
Viremia rate (% ± SE)^*∗*^	15.5 ± 2.2	20.3 ± 5.5	0.41	22.5 ± 3.3	35.1 ± 5.1	**<0.001**	0.34	**0.09**
1st viremia (m post-Tx)	3.5 ± 4.2	4.4 ± 2.8	0.42	8.1 ± 15.2	4.5 ± 5.9	**0.09**	**0.02**	0.94
Peak levels (copies/PCR)	423 ± 734	1801 ± 2278	**0.09**	4549 ± 27703	2919 ± 4658	0.64	0.23	0.28
Duration (m)	0.7 ± 0.5	1.0 ± 0.6	0.16	1.1 ± 1.3	1.8 ± 3.4	0.20	**0.02**	0.15
CMV-PCR > 50 copies/PCR								
Viremia rate (% ± SE)^*∗*^	12.7 ± 2.0	18.6 ± 5.4	0.30	18.7 ± 3.3	35.1 ± 5.1	**<0.001**	0.59	**0.05**
1st viremia (m post-Tx)	3.1 ± 3.1	4.4 ± 3.0	0.27	9.2 ± 16.7	4.5 ± 5.9	**0.06**	**0.01**	0.93
Peak levels (copies/PCR)	496 ± 779	1978 ± 2316	**0.09**	5718 ± 30980	2919 ± 4658	0.52	0.23	0.38
Duration (m)	0.8 ± 0.5	1.1 ± 0.6	0.15	1.2 ± 1.4	1.8 ± 3.4	0.29	**0.03**	0.19

^*∗*^The viremia rates (%  ± standard error [SE]) at 5 years after transplant (Tx) were estimated by the Kaplan-Meier method and the group differences were assessed by the log-rank test. Results for 1st viremia, peak levels, and duration are mean ± standard deviation (SD).

DES: desensitization; post-Tx: posttransplant.

**Table 4:** EBV viremia in EBV sero(+) versus sero(−) patients in the DES and non-DES groups.

	DES group (*n* = 368)			Non-DES group (*n* = 524)			*p* value (DES versus non-DES)	
	EBV sero(+) (*n* = 348)	EBV sero(−) (*n* = 13)	*p* value (sero+ versus −)	EBV sero(+) (*n* = 453)	EBV sero(−) (*n* = 33)	*p* value (sero+ versus −)	In EBV sero(+)	In EBV sero(−)
EBV-PCR > 5.0 copies/PCR								
Viremia rate (% ± SE)^*∗*^	14.4 ± 4.1	16.7 ± 10.8	0.29	32.0 ± 5.3	36.7 ± 9.0	**0.02**	**<0.001**	0.22
1st viremia (m post-Tx)	19.8 ± 20.0	7.1 ± 0.9	**0.01**	14.6 ± 20.8	5.5 ± 5.9	**0.00**	0.29	0.45
Peak levels (copies/PCR)	183 ± 531	29 ± 17	0.18	62 ± 130	160 ± 189	0.14	0.29	**0.06**
Duration (m)	7.0 ± 18.1	3.3 ± 2.8	0.45	4.1 ± 6.2	7.1 ± 6.6	0.21	0.46	0.37
EBV-PCR > 30 copies/PCR								
Viremia rate (% ± SE)^*∗*^	2.8 ± 1.1	8.3 ± 8.0	0.20	11.1 ± 3.3	26.5 ± 8.2	**<0.001**	**<0.01**	0.21
1st viremia (m post-Tx)	15.6 ± 21.2	6.2 ± 0.0	na	11.1 ± 17.3	4.7 ± 6.3	0.12	0.62	na
Peak levels (copies/PCR)	527 ± 817	46 ± 0.0	na	141 ± 185	214 ± 197	0.40	0.25	na
Duration (m)	18.5 ± 27.9	6.0 ± 0.0	na	7.3 ± 7.9	8.0 ± 6.9	0.83	0.33	na
EBV-PCR > 50 copies/PCR								
Viremia rate (% ± SE)^*∗*^	2.5 ± 1.1	0	0.61	5.4 ± 1.7	23.3 ± 7.9	**<0.001**	**0.09**	**0.08**
1st viremia (m post-Tx)	9.4 ± 7.8	na	na	7.1 ± 9.2	5.2 ± 6.6	0.59	0.60	na
Peak levels (copies/PCR)	691 ± 884	na	na	213 ± 214	239 ± 197	0.79	0.28	na
Duration (m)	23.6 ± 30.6	na	na	8.1 ± 8.5	8.6 ± 7.2	0.89	0.31	na
PTLD, *n* (%)	0 (0)	0 (0)	na	0 (0)	0 (0)	na	na	na

^*∗*^The viremia rates (%  ± standard error [SE]) at 5 years after transplant (Tx) were estimated by the Kaplan-Meier method and the group differences were assessed by the log-rank test. Results for 1st viremia, peak levels, and duration are mean ± standard deviation (SD).

DES: desensitization, PTLD: posttransplant lymphoproliferative disorder, and post-Tx: posttransplant.

**Table 5:** CMV and EBV viremia in sero(+) non-DES patients who received valganciclovir (VGCV-LD) versus acyclovir prophylaxis (ACV-anti-IL-2R).

Patients	Number of patients (%)
	w/CMV-PCR > 30 copies/PCR

CMV sero(+) non-DES	
VGCV-LD (*n* = 176)	31 (17.6)
ACV-anti-IL-2R (*n* = 175)	26 (14.9)

VGCV-LD-ATZ (*n* = 25)	7 (28.0)
VGCV-LD-ATG (*n* = 151)	24 (15.9)

	w/EBV-PCR > 30 copies/PCR

EBV sero(+) non-DES	
VGCV-LD (*n* = 200)	22 (11.0)
ACV-anti-IL-2R (*n* = 215)	6 (2.8)^*∗*^

VGCV-LD-ATZ (*n* = 39)	2 (5.1)
VGCV-LD-ATG (*n* = 161)	20 (12.4)

VGCV: valganciclovir; ACV: acyclovir.

LD: lymphocyte depletion; anti-IL-2R: anti-IL-2 receptor.

ATZ: alemtuzumab; ATG: anti-thymocyte globulin.

^*∗*^*p* < 0.001 versus VGCV-LD by Fisher's exact test.

**Table 6:** Viral infection and allograft rejection (AR) in DES and non-DES patients.

Allograft rejection (AR)	Kidney transplant patients		*p* value^*∗∗*^
	DES group (*n* = 363)	Non-DES group (*n* = 497)	
AR, *n* (%)			
Any AR	68 (18.7)	70 (14.1)	**0.07**
ABMR	44 (12.1)	11 (2.2)	**<0.001**
CMR	41 (11.3)	68 (13.7)	0.35
CMV, EBV, or BKV viremia^*∗*^, *n* (%)	80 (22.0)	138 (27.8)	**0.06**
AR within 6 months after VI, *n* (%)			
Any AR after VI	15/80 (18.8)	16/138 (11.6)	0.16
ABMR after VI	8/80 (10.0)	3/138 (2.2)	**0.02**
CMR after VI	11/80 (13.8)	15/138 (10.9)	0.52

^*∗*^Viremia (VI) with peak levels > 50 copies/PCR for CMV & EBV and >2500 copies/ml for BKV viremia was analyzed.

^*∗∗*^The comparison between the two groups was performed by Fisher's exact test.
